# Aneurysmal degeneration of fluoropolymer-coated paclitaxel-eluting stent in the superficial femoral artery: a rising concern

**DOI:** 10.1186/s42155-021-00245-3

**Published:** 2021-07-03

**Authors:** Takuya Tsujimura, Osamu Iida, Mitsutoshi Asai, Masaharu Masuda, Shin Okamoto, Takayuki Ishihara, Kiyonori Nanto, Takashi Kanda, Yasuhiro Matsuda, Yosuke Hata, Hiroyuki Uematsu, Taku Toyoshima, Naoko Higashino, Toshiaki Mano

**Affiliations:** Kansai Rosai Hospital Cardiovascular Center, 3-1-69 Inabaso Amagasaki, Hyogo, 660-8511 Japan

**Keywords:** Endovascular therapy, Fluoropolymer-coated paclitaxel-eluting stent, Aneurysmal degeneration

## Abstract

**Background:**

Although several clinical reports demonstrated a durable patency rate after a novel fluoropolymer-coated paclitaxel-eluting stent (Eluvia; Boston Scientific, Marlborough, MA, USA) placement, aneurysmal degeneration after drug-eluting stent (Eluvia) placement has raised clinical concerns. Here, we report a case with progressive aneurysm formation on serial angiography and intravascular ultrasound over 50 months after drug-eluting stent (Eluvia) placement for a superficial femoral artery atheromatous plaque.

**Case presentation:**

A 79-year-old woman with right leg intermittent claudication at 100 m distance was referred to our hospital. Pre-procedural angiography showed long-segment severe stenosis from the middle-to-distal part of the right superficial femoral artery, and a 7 mm wide drug-eluting stent (Eluvia) was placed. However, the patient had a recurrence of intermittent claudication in the right lower extremity 25 months thereafter. Angiography revealed de novo stenosis in the distal part of the popliteal artery and proximal superficial femoral artery in-stent restenosis. Subsequently, the patient underwent endovascular therapy for these lesions. In addition, intravascular ultrasound at the time of endovascular therapy revealed femoral artery enlargement with a maximum vessel diameter of 10.0 mm at the distal edge of the stent. Intermittent claudication on the right side recurred again 50 months after drug-eluting stent (Eluvia). Angiography demonstrated de novo severe stenosis from the distal part of the superficial femoral artery to the middle part of the popliteal artery. Peri-stent contrast staining was found at the distal part of the drug-eluting stent (Eluvia) site. Intravascular ultrasound showed a further enlargement of maximum vessel diameter to 12.0 mm at the distal edge of the stent.

**Conclusions:**

We report a case with progressive aneurysm degeneration on serial angiography and intravascular ultrasound over 50 months after drug-eluting stent (Eluvia) placement for a superficial femoral artery stenosis.

## Background

With the development of anti-restenotic devices, endovascular therapy (EVT) has become the first-line treatment for femoropopliteal lesions (Aboyans et al, 2018; Conte et al, 2015; Gerhard-Herman et al, 2017). The IMPERIAL randomized trial compared a novel fluoropolymer-coated paclitaxel-eluting stent (Eluvia; Boston Scientific, Marlborough, MA, USA) with a polymer-free paclitaxel-coated stent (Zilver PTX; Cook Corporation, Bloomington, IN, USA), demonstrating a durable patency rate after drug-eluting stent (Eluvia) placement (Gray et al. 2018; Müller-Hülsbeck et al. 2021). However, the problem of aneurysmal degeneration after drug-eluting stent (Eluvia) placement has been raised as a clinical issue in several reports (Gray et al. 2018; Müller-Hülsbeck et al. 2021; Bisdas et al. 2018). Here, we report a case with progressive aneurysm formation on serial angiography and intravascular ultrasound over 50 months after drug-eluting stent (Eluvia) placement for a superficial femoral artery (SFA) atheromatous plaque.

## Case presentation

**Fig. 1:**
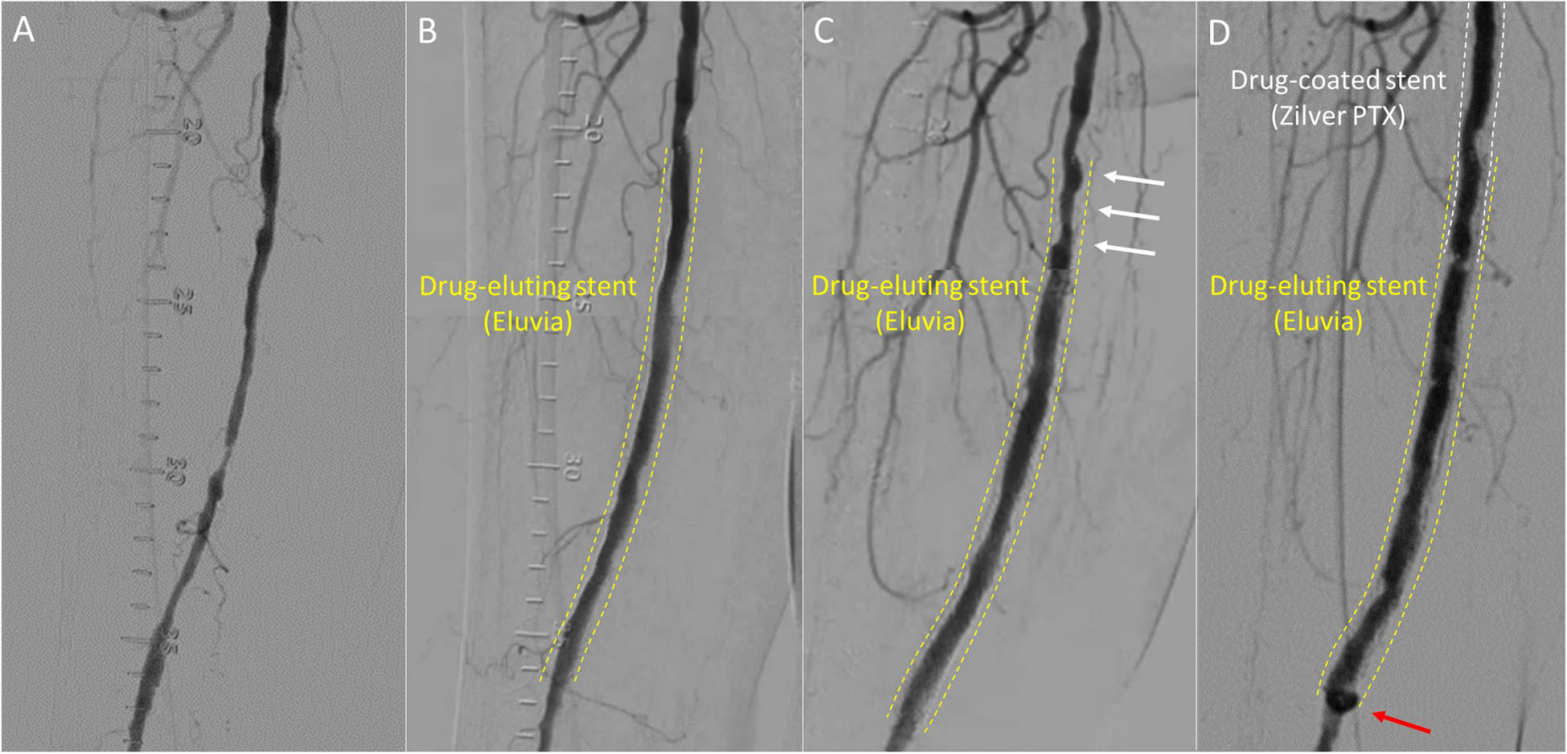
Conventional angiogram. **A**: Initial angiography. Initial angiography revealing a severe stenosis from the middle-to-distal part of the right superficial femoral artery. **B**: Angiography immediately after drug-eluting stent (Eluvia) placement. Angiography showing optimal expansion after drug-eluting stent (Eluvia) placement. **C**: Angiography 25 months after drug-eluting stent (Eluvia) placement. Angiography revealing proximal in-stent restenosis at the drug-eluting stent (Eluvia) placement site (white arrows). **D**: Angiography 50 months after drug-eluting stent (Eluvia) placement. Angiography documenting peri-stent contrast staining at the distal part of the drug-eluting stent (Eluvia) placement site (red arrow)

**Fig. 2:**
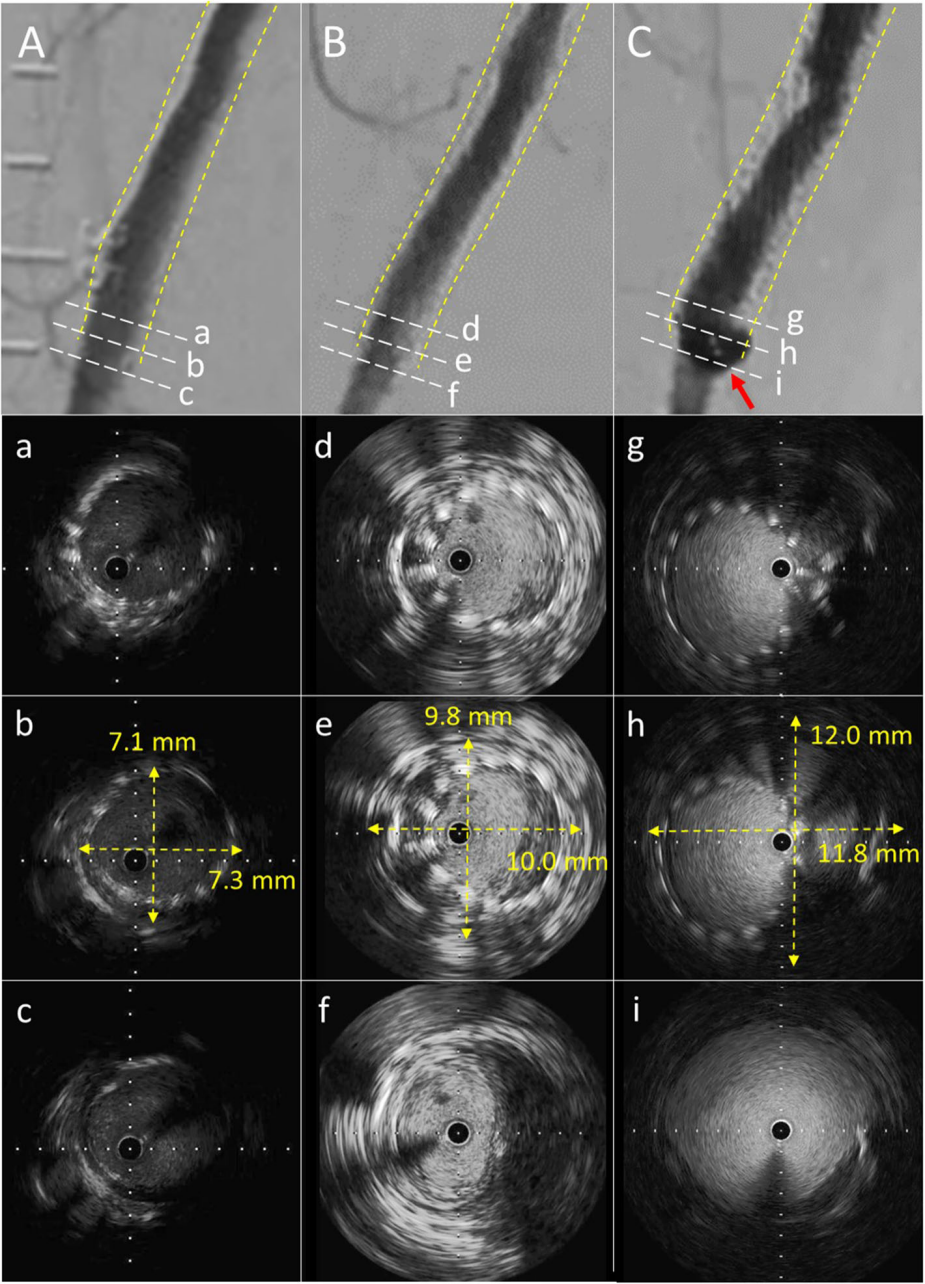
Conventional angiogram and intravascular ultrasound (IVUS) immediately, 25 months, and 50 months after drug-eluting stent (Eluvia) placement. A: Angiography immediately after drug-eluting stent (Eluvia) placement. Angiography showing optimal expansion. B: Angiography 25 months after drug-eluting stent (Eluvia) placement. Angiography showing no significant stenosis or peri-stent contrast staining in the distal part of the right superficial femoral artery. C: Angiography 50 months after drug-eluting stent (Eluvia) placement. Angiography demonstrating peri-stent contrast staining in the distal part of the drug-eluting stent (Eluvia) placement site (red arrow). a-c: Images of IVUS immediately after drug-eluting stent (Eluvia) placement. IVUS demonstrating optimal expansion of drug-eluting stent (Eluvia) (a-c). Maximum vessel diameter was 7.3 mm at the distal edge of the drug-eluting stent (Eluvia) placement site (b). d-f: Images of IVUS 25 months after drug-eluting stent (Eluvia) placement. IVUS revealing vessel enlargement with a maximum vessel diameter of 10.0 mm at the distal edge of the drug-eluting stent (Eluvia) placement site (d-f). g-i: Images of IVUS 50 months after drug-eluting stent (Eluvia) placement. IVUS showing further enlargement of the maximum vessel diameter to 12.0 mm at the distal edge of the drug-eluting stent (Eluvia) placement site (g-i). Enlargement of the lumen and stent malapposition (g and h)

**Fig. 3:**
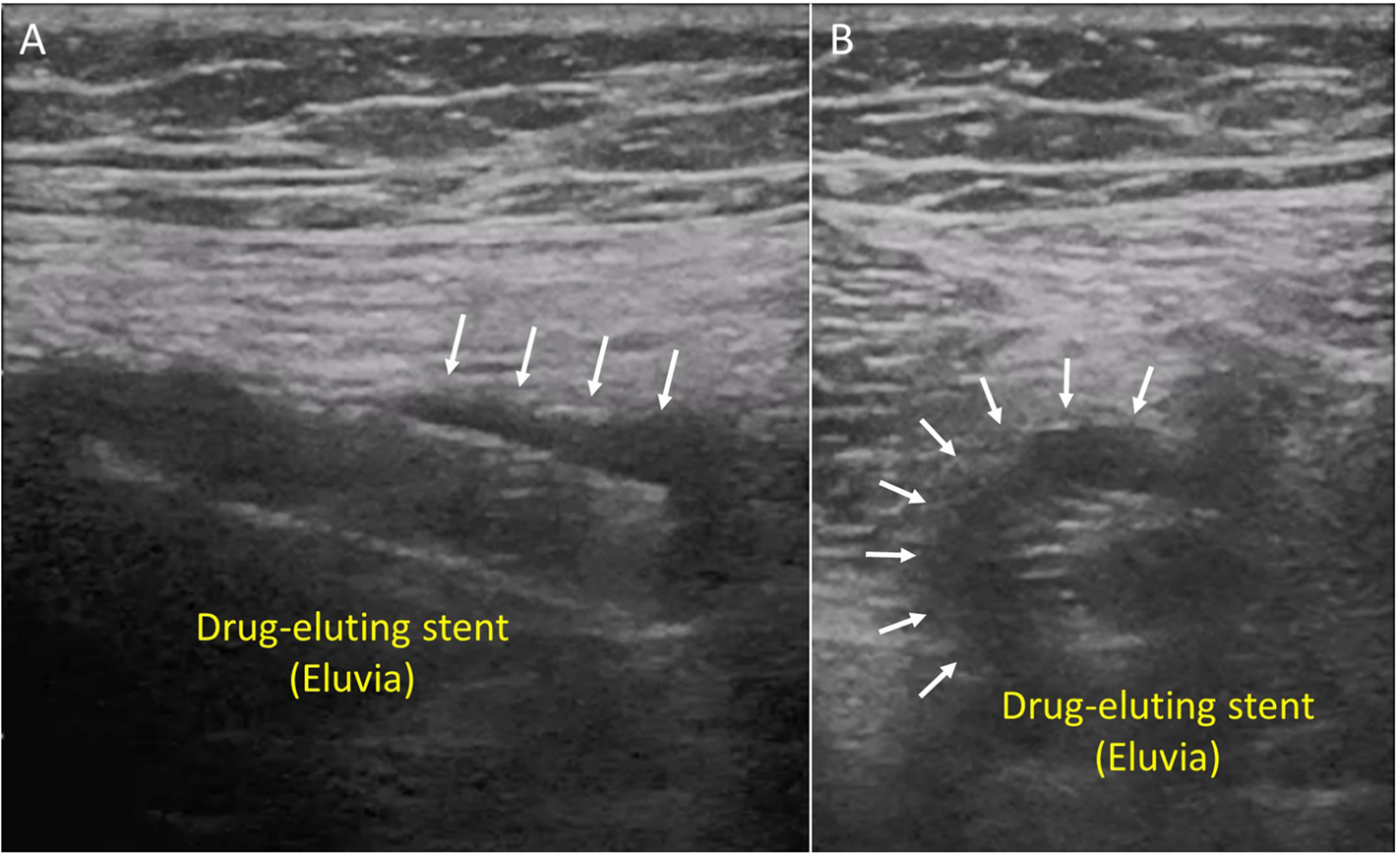
Duplex images 50 months after drug-eluting stent (Eluvia) placement. Duplex images showing a hypoechogenic halo around the distal edge of the drug-eluting stent (Eluvia) (**a** and **b**)

A 79-year old woman with claudication in the right lower extremity which decreased her quality of life was referred to our hospital. Past medical history included hypertension, dyslipidemia and coronary artery disease. The ankle brachial index (ABI) on the right side was 0.71. Pre-procedural angiography showed severe stenosis from the middle-to-distal part of the right SFA and a drug-eluting stent (Eluvia, 7.0 × 150 mm) was placed without pre-dilation because the lesion was not severe calcified lesion. After stent placement, we performed adequate post-dilation, resulting in the optimal expansion of drug-eluting stent (Eluvia) (Fig. 1A, B). After drug-eluting stent (Eluvia) placement, the ABI increased from 0.71 to 0.85 and the patient’s symptoms improved. Since then, she has been taking dual antiplatelet therapy with aspirin (100 mg/day) and clopidogrel (75 mg/day). However, she had a recurrence of intermittent claudication in the right lower extremity 25 months after drug-eluting stent (Eluvia) placement. Angiography revealed de novo stenosis in the distal part of the popliteal artery and proximal in-stent restenosis at the drug-eluting stent (Eluvia) placement site (Fig. 1C). Subsequently, the patient underwent endovascular therapy (EVT) with plain balloon angioplasty for severe stenosis in the distal part of the popliteal artery and a drug-coated stent (Zilver PTX, 7.0 × 120 mm) was placed for the proximal in-stent restenosis of the drug-eluting stent (Eluvia). A completion angiogram showed an optimal angiographic result. In addition, intravascular ultrasound (IVUS, AltaView; Terumo, Tokyo, Japan) at the time of EVT revealed vessel enlargement with a maximum vessel diameter of 7.3–10.0 mm at the distal edge of the drug-eluting stent (Eluvia) placement site (Fig. 2a-f). Thereafter, the patient was symptom-free until intermittent claudication on the right side recurred 50 months after drug-eluting stent (Eluvia) placement. Angiography demonstrated de novo severe stenosis from the distal part of the SFA to the middle part of the popliteal artery. The popliteal artery lesion was dilated using a drug-coated balloon (INPACT Admiral, 4.0 × 80 mm; Medtronic plc., Santa Rosa, CA, USA) and the distal SFA lesion was dilated using a drug-coated balloon (INPACT Admiral, 6.0 × 120 mm), resulting in an adequate angiographic result. However, peri-stent contrast staining (PSS) was found by angiography at the distal part of the drug-eluting stent (Eluvia) placement site (Fig. 1D). IVUS showed a further enlargement of maximum vessel diameter to 12.0 mm at the distal edge of the drug-eluting stent (Eluvia) (Fig. 2g-i). Moreover, enlargement of the lumen and stent malapposition were also found, suggesting progressive aneurysmal degeneration 50 months after drug-eluting stent (Eluvia) placement. Similarly, duplex images showed a hypoechogenic halo suggestive of aneurysmal degeneration around the distal edge of the drug-eluting stent (Eluvia) (Fig. 3).

## Discussion

Here, we report a patient with progressive aneurysmal degeneration assessed by serial angiography and IVUS 50 months after drug-eluting stent (Eluvia) placement for an SFA lesion. Although several clinical studies reported instances of aneurysmal degeneration 12 or 24 months after drug-eluting stent (Eluvia) placement (Gray et al. 2018; Müller-Hülsbeck et al. 2021; Bisdas et al. 2018), there have been no reports of complications with progressive aneurysmal degeneration assessed by serial imaging device beyond 24 months after implantation. To the best of our knowledge, this is the first report showing progressive aneurysmal degeneration assessed by serial angiography and IVUS 50 months after drug-eluting stent (Eluvia) placement.

In coronary arteries, PSS, that is defined as contrast staining outside the stent contour extending to ≥20% of the stent diameter after drug-eluting stent placement, is rare and has a reported incidence ranging from 1.9% to 2.2% (Imai et al. 2011; Tokuda et al. 2016). PSS could be regarded as representing an abnormal response of the vessel wall caused by drug-eluting stent. Several mechanisms have been proposed to account for the development of PSS, including delayed re-endothelialization, inflammatory changes in the medial wall, and hypersensitivity reactions to drugs and polymers (Virmani et al. 2002; Virmani et al. 2004; Joner et al. 2006).

Although the 12-month duplex ultrasound examination in the IMPERIAL randomized trial reported aneurysmal degeneration in only 1.9% (6/309) of patients after drug-eluting stent (Eluvia) placement (Gray et al. 2018), this rose to 8.1% (8/62) in the 12-month results of the Munster registry, thus inconsistent across studies (Bisdas et al. 2018). In one of these cases, immunohistological analysis of the arterial wall revealed an infiltration of CD3^+^, CD56^+^ T cells (Bisdas et al. 2018). Although the exact mechanisms responsible for aneurysmal degeneration after drug-eluting stent (Eluvia) placement are not known, as with coronary arteries, vascular inflammation might be associated with this syndrome.

Drug-eluting stent (Eluvia) is coated with the primer polymer poly (n-butyl methacrylate) and an active layer composed of the matrix polymer poly (vinylidene fluoride-co-hexafluoropropylene). These coatings permit the elution of paclitaxel over approximately 12 months at a dose density of 0.167 μg paclitaxel per mm^2^ stent surface area (Gray et al. 2018). Farb et al. showed a dose-dependent increase in fibrin deposition and medial necrosis after deployment of paclitaxel-eluting stents in rabbit iliac arteries (Farb et al. 2001). Another previous pathological report mentioned that the features of paclitaxel-eluting stent placement included excessive para-strut fibrin deposition and incomplete stent apposition in patients with late stent thrombosis, suggesting that paclitaxel itself was responsible (Nakazawa et al. 2011). In fact, although drug-coated stent (Zilver PTX) is a paclitaxel-coated stent without polymer, some clinical reports also recorded cases with PSS on angiography or extra stent lumen enlargement on serial optical coherence tomography after drug-coated stent (Zilver PTX) placement at later times (Ikeoka et al. 2018; Tomoi et al. 2015). Therefore, it is possible that paclitaxel itself similarly induces vascular inflammation after drug-eluting stent (Eluvia) placement and may contribute to aneurysmal degeneration. It has been reported that the polymer coatings could also cause increased vessel inflammation, which is sometimes severe, even leading to medial disruption and aneurysm (van der Giessen et al. 1996; Byrne et al. 2009; John et al. 2008). Pathological studies regarding vascular responses to drug-coated stent (Zilver PTX) versus drug-eluting stent (Eluvia) showed higher inflammatory reactions around struts in the latter 1-, 3-, 6- and 12-months after placement, possibly caused by continuous exposure to paclitaxel, or the presence of the durable polymer coating (Sakamoto et al. 2021). Furthermore, 24-month results of the IMPERIAL randomized trial showed hypoechogenic halo suggestive of aneurysmal degeneration in 33.7% of patients after drug-eluting stent (Eluvia) placement and 21.4% after drug-coated stent (Zilver PTX) placement, but this difference was not statistically significant (Müller-Hülsbeck et al. 2021). The frequency of aneurysmal degeneration 24 months after drug-eluting stent (Eluvia) placement increased compared to what was seen at 12 months (Gray et al. 2018; Müller-Hülsbeck et al. 2021). Thus, although there was no significant difference, the frequency of occurrence of aneurysmal degeneration tended to be greater for drug-eluting stent (Eluvia) than drug-coated stent (Zilver PTX) (Müller-Hülsbeck et al. 2021). Judging from these results, the durable polymer employed by drug-eluting stent (Eluvia) might be associated with more vessel inflammation, resulting in aneurysmal degeneration at later times. According to the manufacturers of the stent platforms, the constant outward self-expanding force is greater in drug-eluting stent (Eluvia) than drug-coated stent (Zilver PTX), which itself might contribute to vessel inflammation,

Although data on the clinical parameters of patients with aneurysmal degeneration in PAD are scarce, a previous report on coronary artery disease showed that PSS found within 12 months after drug-eluting stent (Eluvia) placement was potentially associated with subsequent target lesion revascularization and very late stent thrombosis (Imai et al. 2011). Bisdas et al. reported that 5 of 62 patients treated by drug-eluting stent (Eluvia) placement had aneurysmal degeneration and one of these presented with clinical worsening caused by the occlusion of the stent (Bisdas et al. 2018). Given these data, long-term follow-up should be mandatory for patients receiving drug-eluting stent (Eluvia) placement. Further investigation is warranted to evaluate the impact of aneurysmal degeneration after drug-eluting stent (Eluvia) placement on clinical outcomes. Although dual antiplatelet therapy is recommended for at least 60 days after drug-eluting stent (Eluvia) placement, its prolongation might be worth considering in patients with aneurysmal degeneration.

## Conclusions

We report a case with progressive aneurysm degeneration on serial angiography and intravascular ultrasound over 50 months after drug-eluting stent (Eluvia) placement for a SFA stenosis.

## Data Availability

Not applicable.

## Abbreviations

ABI: Ankle brachial index
EVT: Endovascular therapy
IVUS: Intravascular ultrasound
PSS: Peri-stent contrast staining
SFA: Superficial femoral artery

## References

[CR1] Aboyans V, Ricco JB, Bartelink MEL, Björck M, Brodmann M, Cohnert T, Collet JP, Czerny M, De Carlo M, Debus S, Espinola-Klein C, Kahan T, Kownator S, Mazzolai L, Naylor AR, Roffi M, Röther J, Sprynger M, Tendera M, Tepe G, Venermo M, Vlachopoulos C, Desormais I, ESC Scientific Document Group (2018). 2017 ESC guidelines on the diagnosis and treatment of peripheral arterial diseases, in collaboration with the European Society for Vascular Surgery (ESVS): document covering atherosclerotic disease of extracranial carotid and vertebral, mesenteric, renal, upper and lower extremity arteries endorsed by: the European stroke organization (ESO) the task force for the diagnosis and treatment of peripheral arterial diseases of the European Society of Cardiology (ESC) and of the European Society for Vascular Surgery (ESVS). Eur Heart J.

[CR2] Bisdas T, Beropoulis E, Argyriou A, Torsello G, Stavroulakis K (2018). 1-year all-comers analysis of the eluvia drug-eluting stent for long Femoropopliteal lesions after suboptimal angioplasty. JACC Cardiovasc Interv.

[CR3] Byrne RA, Joner M, Kastrati A (2009). Polymer coatings and delayed arterial healing following drug-eluting stent implantation. Minerva Cardioangiol.

[CR4] Conte MS, Pomposelli FB, Clair DG, Geraghty PJ, McKinsey JF, Mills JL, Moneta GL, Murad MH, Powell RJ, Reed AB, Schanzer A, Sidawy AN (2015). Society for Vascular Surgery practice guidelines for atherosclerotic occlusive disease of the lower extremities: Management of asymptomatic disease and claudication. J Vasc Surg.

[CR5] Farb A, Heller PF, Shroff S, Cheng L, Kolodgie FD, Carter AJ, Scott DS, Froehlich J, Virmani R (2001). Pathological analysis of local delivery of paclitaxel via a polymer-coated stent. Circulation.

[CR6] Gerhard-Herman MD, Gornik HL, Barrett C, Barshes NR, Corriere MA, Drachman DE, Fleisher LA, Fowkes FGR, Hamburg NM, Kinlay S, Lookstein R, Misra S, Mureebe L, Olin JW, Patel RAG, Regensteiner JG, Schanzer A, Shishehbor MH, Stewart KJ, Treat-Jacobson D, Walsh ME (2017). 2016 AHA/ACC guideline on the Management of Patients with Lower Extremity Peripheral Artery Disease: executive summary: a report of the American College of Cardiology/American Heart Association task force on clinical practice guidelines. J Am Coll Cardiol.

[CR7] Gray WA, Keirse K, Soga Y, Benko A, Babaev A, Yokoi Y, Schroeder H, Prem JT, Holden A, Popma J, Jaff MR, Diaz-Cartelle J, Müller-Hülsbeck S (2018). IMPERIAL investigators. A polymer-coated, paclitaxel-eluting stent (eluvia) versus a polymer-free, paclitaxel-coated stent (Zilver PTX) for endovascular femoropopliteal intervention (IMPERIAL): a randomised, non-inferiority trial. Lancet.

[CR8] Ikeoka K, Okayama K, Watanabe T, Nanto S, Sakata Y, Hoshida S (2018). Refractory Vascular Wall healing after paclitaxel-coated Nitinol stent implantation in the Femoropopliteal artery: a high-resolution Angioscopic assessment. Ann Vasc Dis.

[CR9] Imai M, Kadota K, Goto T, Fujii S, Yamamoto H, Fuku Y, Hosogi S, Hirono A, Tanaka H, Tada T, Morimoto T, Shiomi H, Kozuma K, Inoue K, Suzuki N, Kimura T, Mitsudo K (2011). Incidence, risk factors, and clinical sequelae of angiographic peri-stent contrast staining after sirolimus-eluting stent implantation. Circulation.

[CR10] John MC, Wessely R, Kastrati A, Schömig A, Joner M, Uchihashi M, Crimins J, Lajoie S, Kolodgie FD, Gold HK, Virmani R, Finn AV (2008). Differential healing responses in polymer- and nonpolymer-based sirolimus-eluting stents. JACC Cardiovasc Interv.

[CR11] Joner M, Finn AV, Farb A, Mont EK, Kolodgie FD, Ladich E, Kutys R, Skorija K, Gold HK, Virmani R (2006). Pathology of drug-eluting stents in humans: delayed healing and late thrombotic risk. J Am Coll Cardiol.

[CR12] Müller-Hülsbeck S, Benko A, Soga Y, Fujihara M, Iida O, Babaev A, O'Connor D, Zeller T, Dulas DD, Diaz-Cartelle J, Gray WA (2021). Two year efficacy and safety results from the IMPERIAL randomized study of the eluvia polymer-coated drug-eluting stent and the Zilver PTX polymer-free drug-coated stent. Cardiovasc Intervent Radiol.

[CR13] Nakazawa G, Finn AV, Vorpahl M, Ladich ER, Kolodgie FD, Virmani R (2011). Coronary responses and differential mechanisms of late stent thrombosis attributed to first-generation sirolimus- and paclitaxel-eluting stents. J Am Coll Cardiol.

[CR14] Sakamoto A, Torii S, Jinnouchi H, Fuller D, Cornelissen A, Sato Y, Kuntz S, Mori M, Kawakami R, Kawai K, Fernandez R, Paek KH, Gadhok N, Guo L, Kolodgie FD, Young B, Ragheb A, Virmani R, Finn AV (2021) Vascular response of a polymer-free, paclitaxel-coated stent (Zilver PTX) versus a polymer-coated, paclitaxel-eluting stent (eluvia) in healthy swine Femoropopliteal arteries. J Vasc Interv Radiol. 10.1016/j.jvir.2021.02.014 10.1016/j.jvir.2021.02.014 33677117

[CR15] Tokuda T, Yamawaki M, Takahara M, Mori S, Makino K, Honda Y, Takafuji H, Takama T, Tsutsumi M, Sakamoto Y, Takimura H, Kobayashi N, Araki M, Hirano K, Ito Y (2016). Comparison of long-term clinical outcomes of lesions exhibiting focal and segmental Peri-stent contrast staining. J Am Heart Assoc.

[CR16] Tomoi Y, Kuramitsu S, Soga Y, Aihara H, Ando K, Nobuyoshi M (2015). Vascular response after Zilver PTX stent implantation for superficial femoral artery lesions: serial optical coherence tomography findings at 6 and 12 months. J Endovasc Ther.

[CR17] van der Giessen WJ, Lincoff AM, Schwartz RS, van Beusekom HM, Serruys PW, Holmes DR, Ellis SG, Topol EJ (1996). Marked inflammatory sequelae to implantation of biodegradable and nonbiodegradable polymers in porcine coronary arteries. Circulation.

[CR18] Virmani R, Guagliumi G, Farb A, Musumeci G, Grieco N, Motta T, Mihalcsik L, Tespili M, Valsecchi O, Kolodgie FD (2004). Localized hypersensitivity and late coronary thrombosis secondary to a sirolimus-eluting stent: should we be cautious?. Circulation.

[CR19] Virmani R, Liistro F, Stankovic G, Di Mario C, Montorfano M, Farb A, Kolodgie FD, Colombo A (2002). Mechanism of late in-stent restenosis after implantation of a paclitaxel derivate-eluting polymer stent system in humans. Circulation.

